# The relationship between family functioning and social media addiction among university students: a moderated mediation model of depressive symptoms and peer support

**DOI:** 10.1186/s40359-024-01818-2

**Published:** 2024-06-10

**Authors:** Yating Qi, Miaomiao Zhao, Tingting Geng, Ziqi Tu, Qingyun Lu, Ruyu Li, Luyao Niu, Wenjie Qu, Yaqin Zhong, Yuexia Gao

**Affiliations:** 1 Department of Health Management, School of Public Health, Nantong University, 9 Seyuan Road, Nantong, Jiangsu 226019 China; 2 Institute for Health Development, Nantong University, Nantong, China; 3 Department of Clinical Medicine, The First Clinical College of Southern Medical University, Guangzhou, China

**Keywords:** Social media addiction, Family functioning, Peer support, Depressive symptoms, University students

## Abstract

**Background:**

Social media addiction (SMA) is an increasing problem, especially among young adults. Little is known about university students’ SMA and family functioning. This study aimed to explore the mediating effect of depressive symptoms and the moderating effect of peer support in the relationship between family functioning and SMA among young adults.

**Methods:**

A sample of 1862 Chinese university students completed an online survey including the Bergen Social Media Addiction Scale (BSMAS), Family APGAR, the Patient Health Questionnaire (PHQ-9), peer support, and demographic characteristics. Hierarchical regression and moderated mediation analysis were used to test the effects and pathways among them.

**Results:**

Of the 1840 participants, 30.11% experienced SMA, 38.80% had family dysfunction and 15.98% had depressive symptoms. Hierarchical multiple regression showed better family functioning significantly predicted less SMA (*β* = -0.26, *p* < 0.001) and lower depressive symptoms (*β* = -0.58, *p* < 0.001), after adjusting for covariates. Mediation analysis verified that depressive symptoms mediated the effect of family functioning on SMA (indirect effect = -0.22, 95%CI[-0.28, -0.17]). Furthermore, the interaction of family functioning and peer support was negatively related to depressive symptoms (*β*= -0.03, 95% CI[-0.05, -0.01]) and the interaction of depressive symptoms and peer support was positively related to SMA (*β* = 0.01, 95%CI[0.004, 0.02]). Additional analysis further confirmed that peer support decreased depressive symptoms among young adults from dysfunctional families, and increased SMA behaviors in individuals with depressive symptoms.

**Conclusion:**

Better family functioning and lower depressive symptoms may contribute to less social media addiction among Chinese university students. Peer support could moderate the mediating role of depressive symptoms on social media addiction in individuals with family dysfunction.

**Supplementary Information:**

The online version contains supplementary material available at 10.1186/s40359-024-01818-2.

## Introduction

Social media, with its great popularity and strong functioning, has become an indispensable aspect of daily life [1]. According to the 51st Statistical Report on Internet Development in China [2], 32.9% of Internet users were adolescents and youth under 30 years of age. A national survey among 5118 college students revealed that 99.39% of them use social media and spend more than 4 h on social media daily in China [3]. As social media usage increases, it may give rise to negative outcomes [4], such as overreliance and addictive behavior [5] on social media among young adults.

Social media addiction (SMA), defined as prolonged exposure to and repeated use of social media [6], is characterized by a dysfunctional form of being unable to resist the motivation to follow social media, and experiencing distress if prohibited from using social media [7]. A meta-analysis of 64 countries demonstrated that the pooled prevalence rate of SMA was 17.42% in the general population [8]. In China, 15.2% of university students have SMA [9]. Current evidence derived from cross-sectional studies and meta-analyses demonstrated that SMA may be related to negative consequences, like poor academic performance [10], sleep disorders [11], worse psychological well-being [12], and so on [13]. Therefore, SMA may be a more noteworthy public health concern, and it is vital to explore the risk factors and potential mechanisms of SMA.

Ecological system theory [14] emphasizes that the interaction between individual characteristics and ecological systems (e.g., family and society) shapes the developmental outcomes of individuals. Although ecological system theory and existing studies [15, 16] have indicated that the formation of SMA is strongly affected by the structures and interactions of various systems, few studies have examined the interactive effects of family functioning, peer support, and depressive symptoms on SMA among Chinese university students.

### Family functioning and SMA

According to the McMaster Family Functioning Model Theory, family functioning refers to providing an appropriate environment and promoting the growth and development of family members, including physical, psychological, social, and other domains [17]. Previous studies have suggested that good family functioning involving adaptation, interaction, cohesion, closeness, response, and problem-solving [18] between family members is essential for lowering the likelihood of psychological distress [19] and problematic behavior [20]. For example, a longitudinal study involving 3 waves of follow-up in Hong Kong among 3325 junior high school students revealed that good family functioning was related to less SMA [21]. Family dysfunction (e.g., poor relationships and intense conflict) was identified to be associated with increased SMA significantly [22]. However, another recent longitudinal study among 1344 middle school students found that family functioning was not related to addiction to social media [23]. In this afore-mentioned study, SMA was measured by one single item “how much time spent on social media ”, which evaluated one aspect of SMA, resulting in oversimplified and controversial findings. Thus, no consensus has been reached regarding the relationship between family functioning and SMA. Furthermore, studies on the relationship between family functioning and SMA have mainly focused on children and adolescents, while few studies have been conducted on university students in China. Therefore, the relationship between family functioning and SMA among Chinese university students needs to be studied further.

### Depressive symptoms as a mediator

Depressive symptoms, as the main factor contributing to the worldwide burden of disease, have a prevalence rate of 33.60% among Chinese university students [24]. On the one hand, depressive symptoms were reported to be directly related to problematic behaviors, including e-cigarette use [24] and problematic smartphone use [25]. A meta-analysis of 50 studies with 27,935 participants concluded that there was a positive correlation between depressive symptoms and SMA [26]. Another study involving 8,912 university students from seven countries highlighted that depressive symptoms were positively related to SMA [27]. On the other hand, previous research suggested that family functioning was a predictor of depressive symptoms [28]. Good family functioning facilitates the fulfillment of individuals’ relatedness needs [29] and subsequently enhances physical and mental well-being [30]. A birth cohort over 3 decades demonstrated that family conflict played a key role in increasing adolescent depressive symptoms. According to compensatory Internet use theory [31], people may resort to social media as a coping strategy to make up for unsatisfied offline psychological needs and alleviate negative emotions (e.g., depressive symptoms) during challenging real-life situations (e.g., family dysfunction) [32]. Overall, considering the above theoretical and empirical evidence, good family functioning may decrease individuals’ depressive symptoms and social media addiction. And depressive symptoms were positively related to SMA [33]. Therefore, we hypothesized that depressive symptoms may mediate the association between family functioning and SMA.

### Peer support as a moderator

Peer support, referring to mutual assistance, empathy exchange, and receiving companionship from peers [34], may improve individuals’ psychological state [35] and problematic behavior [36](e.g., SMA [36, 37]). Nevertheless, when social media is considered an expansion of social connections to maintain peer relationships and seek peer assistance, peer support may become a trigger for SMA [38]. In line with a systematic review, peer support had a positive impact on SMA [39]. Thus, the relationship between peer support and SMA should be further explored.

In addition, many studies have shown that peer support plays an important protective role in the associations among life events, mental disorders, and addictive behavior. According to the stress-buffering model, the interaction between positive experience and peer support has the potential to counter the negative effects of stress [40], promote mental health [41], and reduce addictive behavior [42]. For instance, a longitudinal study conducted on 1301 middle school students emphasized that peer support may buffer the effect of family functioning on adolescent depressive symptoms [43]. However, a recent two-year longitudinal study pointed out that higher levels of peer support exacerbated depressive symptoms in adolescents who experienced family violence [44]. Another cross-sectional study conducted on 1258 participants aged 13 to 24 years indicated that better peer support may increase the risk of SMA in individuals suffering from negative emotions [45], indicating that peer support may reinforce the effects of negative events. Based on the afore-mentioned studies, the moderating effect of peer support on the relationships among family functioning, depressive symptoms, and SMA deserves further exploration.

### The present study

**Fig. 1:**
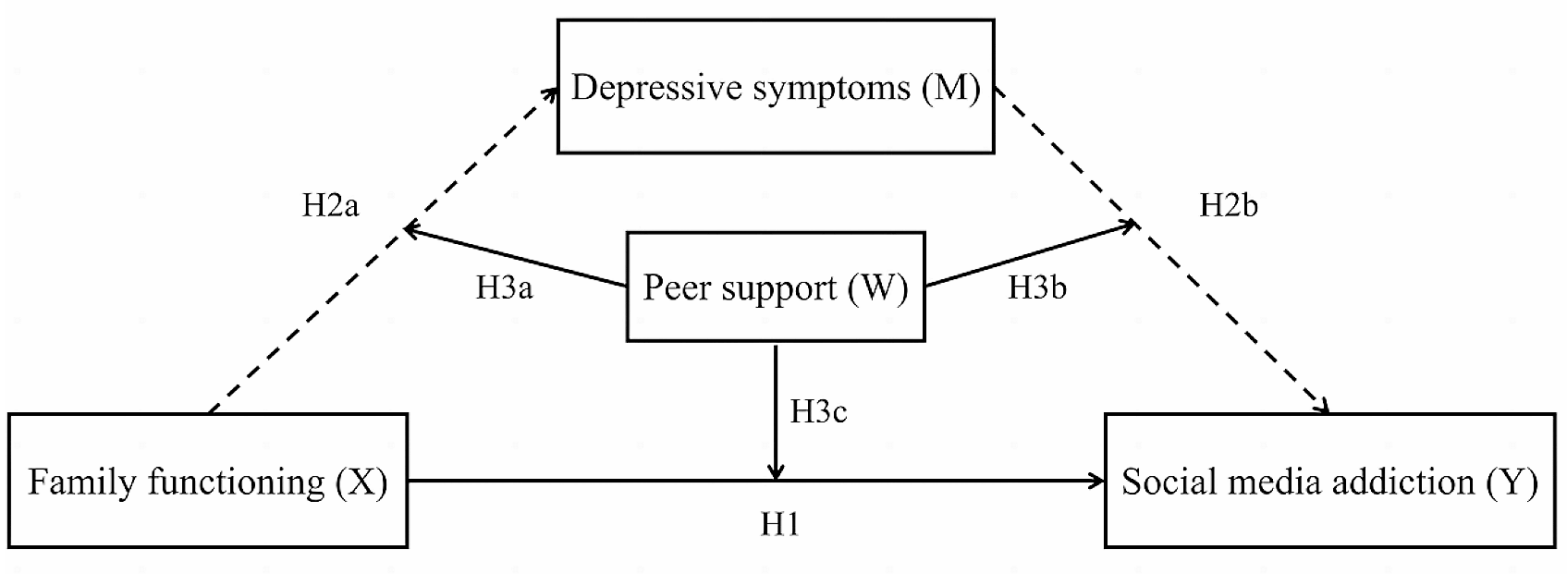
Hypothesized moderated mediation model

Taken together, family functioning, depressive symptoms, and peer support may be correlated to SMA, but there are still some controversial results for the possible mechanisms underlying these associations among Chinese university students. Hence, the current study developed a moderated mediation model (see Fig. 1) to elucidate the pathway from family functioning to SMA by analyzing the mediating effect of depressive symptoms and the moderating effect of peer support on this association among Chinese university students. The following hypotheses were formulated:

#### H1

Good family functioning may be negatively associated with SMA among university students in China.

#### H2

Depressive symptoms may mediate the relationship between family functioning and SMA.

#### H3

Peer support might function as a moderator of the direct effect (H3c: family functioning-SMA) and/ or indirect effect (H3a: family functioning-depressive symptoms; H3b: depressive symptoms-SMA) of family functioning on SMA.

## Methods

### Procedures and participants

The current study was conducted at Nantong University, a comprehensive school in Jiangsu Province in eastern China, from April to May 2020. We used cluster sampling to randomly select two majors from three major categories (science and technology, liberal arts, and medicine) and then selected five classes from freshmen to seniors and above in each major. 12 Counselors from six majors, who provided guidance, counseling, and advocacy services for these young adults at Nantong University, were recruited as investigators and informed about the general aim of the research and the procedure of data collection. A total of 1862 university students completed the questionnaire through an online survey platform “Questionnaire Star” (https://www.wjx.cn).

The inclusion criteria of participants were as follows: (1) full-time university students aged 18 to 25; (2) could use social media daily, including WeChat, Weibo, Facebook, and so on. The exclusion criteria included (1) less than 18 years old; (2) suffering from mental illness; (3) having visual disorders. The respondents were also told the aim of the study and provided informed consent electronically. Participants giving none or partial responses were deleted from the data set, and 1840 valid samples were analyzed, for an effective response of 98.82%.

### Measures

#### Predictor variable

Family functioning was measured using the Family Affection Partnership Growth Adaptation Resolve Scale [46], which uses a 3-point Likert scale to score each item from 0 (nearly none) to 2 (almost always). The responses of the five items were summed to generate a total score, with a higher score indicating better family functioning. A total score below 4 reflects a very dysfunctional family, a score between 4 and 6 reflects a moderately dysfunctional family and a score ranging from 7 to 10 reflects a well-functioning family [47]. The internal consistency dependability of this study was high with a Cronbach’s alpha coefficient of 0.903.

#### Mediating variable

The severity of depressive symptoms was examined using the Patient Health Questionnaire (PHQ-9) [48], which measures the extent of depressive symptoms on a 4-point Likert scale ranging from 0 (not at all) to 3 (more than a week). The sum of the scores ranged from 0 to 27, and individuals who scored ≥ 10 points were classified as suffering from depressive symptoms [49]. The Cronbach’s alpha coefficient obtained was 0.929.

#### Moderating variable

Peer support was evaluated using six items from the Healthy Lifestyle Scale for University Students (HLSUS) [50], which is a self-administered instrument that consists of 8 dimensions, and each item was evaluated using a 5-point Likert scale ranging from 1 to 5, with a higher total score signifying a higher level of peer support. The Cronbach’s alpha coefficient was 0.924.

#### Dependent variable

The SMA was evaluated by the Bergen Social Media Addiction Scale (BSMAS) [51], which measures salience, emotion regulation, tolerance, withdrawal, conflict, and relapse, with scores ranging from 1 to 5. The cumulative score is determined by summing the scores of the six items, spanning a range of 6 to 30. Individuals with a score of 19 or higher [52] were considered to have SMA. The Bergen Social Media Addiction Scale has been widely applied in Chinese university students and has great psychometric properties with high internal consistency (Cronbach’s α = 0.85), criterion, and construct validity [53]. The Cronbach’s alpha in the current study was 0.889.

#### Covariates

Covariates including age [54], gender [55], grade [56], and major [57] were obtained from participants and included in the analysis, as these variables were identified to be related to SMA in the previous studies. Gender is a dummy variable that equals 1 for males and 2 for females. The grade level was a categorical variable: 1 = freshmen, 2 = sophomores, 3 = juniors, and 4 = seniors and above. Major is a dummy variable: 1 for medical majors, including public health and preventive medicine, and clinical medicine, and 2 for other majors.

### Statistical analyses

All the statistical analyses were carried out using SPSS 27.0 and PROCESS macro. First, descriptive analyses were performed to characterize the sample (see Table 1). Second, partial correlation analysis was performed to test how these variables were related after adjusting for control variables (see Table 2). Third, hierarchical multiple regressions were utilized to examine the mediating impact of depressive symptoms (Table 3). Model 1 tested the connection between family functioning (X) and SMA (Y) (H1). Model 2 tested the connection between family functioning (X) and depressive symptoms (M) (H2). Model 3 examined the direct effect of family functioning (X) and depressive symptoms (M) on the SMA (Y). Model 1 and Model 3 provided direct and indirect paths among these variables (see Table 3). Furthermore, Model 4 of the PROCESS was used to analyze the mediating effect of depressive symptoms (see Table 4) on the relationship between family functioning (X) and SMA (Y).

**Table 1:** Basic characteristics of university students (*n* = 1840)

Variables		*N*(%)	Social media addiction
**Gender**	Male	535(29.08)	148(27.66)
	Females	1305(70.92)	406(31.11)
**Grade**	Freshmen	535(29.08)	143(26.73)
	Juniors	355(19.51)	88(24.51)
	Sophomores	659(35.82)	225(34.14)
	Seniors and above	287(15.59)	98(34.15)
**Major**	Medical field	1134(61.63)	339(29.89)
	Other fields	706(38.37)	215(30.45)
**Only Child**	Yes	994(54.02)	293(29.48)
	No	846(45.98)	261(30.85)
**Family functioning (APGAR score)**	dysfunctional	93(5.05)	44(47.31)
	moderately dysfunctional	621(33.75)	209(33.66)
	well-function	1126(61.20)	301(26.73)
**Depressive symptoms (PHQ-9 score)**	None	1546(84.02)	377(24.39)
	Moderate and severe	294(15.98)	177(60.20)
**Total**		1840(100)	554(30.11)

**Table 2:** Partial correlations among predictor, mediator, moderator, and dependent variables

Variables	Mean ± SD	1	2	3	4
1 Family functioning	7.52 ± 2.48	1			
2 Depressive symptoms	5.51 ± 5.39	-0.27^***^	1		
3 Social media addiction	16.73 ± 5.22	-0.12^***^	0.40^***^	1	
4 Peer support	22.83 ± 4.90	0.36^***^	-0.16^***^	-0.02	1

*Note* The variables of age, gender, grade, and major were controlled. ^***^p < 0.001

**Table 3:** Mediation models of family functioning on social media addiction through depressive symptoms

Variables	Model 1 (Social media addiction)			Model 2 (Depressive symptoms)			Model 3 (Social media addiction)		
	β	t	95%CI	β	t	95%CI	β	t	95%CI
**Predictor**									
Family functioning	-0.26^***^	-5.18	-0.35, -0.16	-0.58^***^	-11.75	-0.67, -0.48	-0.04	-0.74	-0.13,0.06
**Mediator**									
Depressive symptoms							0.38^***^	17.45	0.34,0.43
**Covariates**									
Age	0.13	0.83	-0.18,0.44	-0.23	-1.45	-0.53,0.08	0.22	1.50	-0.07,0.50
Gender	0.33	1.23	-0.20,0.86	-0.93^**^	-3.45	-1.45, -0.40	0.69^**^	2.74	0.20,1,18
Grade	-0.01	-0.06	-0.36,0.34	0.28	1.58	-0.07,0.62	-0.12	-0.71	-0.44,0.20
Major	0.10	1.01	-0.10,0.31	0.01	0.13	-0.19,0.21	0.10	1.04	-0.09,0.29
Adj-R²	0.01			0.08			0.16		
F	6.32^***^			31.31^***^			56.87^***^		

*NoteN* = 1840; The variables of age, gender, grade, and major were controlled. 95% CI = Bootstrap confidence intervals with lower and upper limits. ^*^p < 0.05, ^**^p < 0.01, ^***^p < 0.001

**Table 4:** Bootstrap test of the mediating effects of depressive symptoms

Effect	β	SE	LLCI	ULCI
Total effects	-0.26	0.05	-0.35	-0.16
Direct effect	-0.04	0.05	-0.13	0.06
Total indirect effect	-0.22	0.03	-0.28	-0.17

*NoteN* = 1840. The variables of age, gender, grade, and major were controlled. LLCI represents a lower level of the confidence interval, and ULCI represents the upper level of the confidence interval

**Fig. 2:**
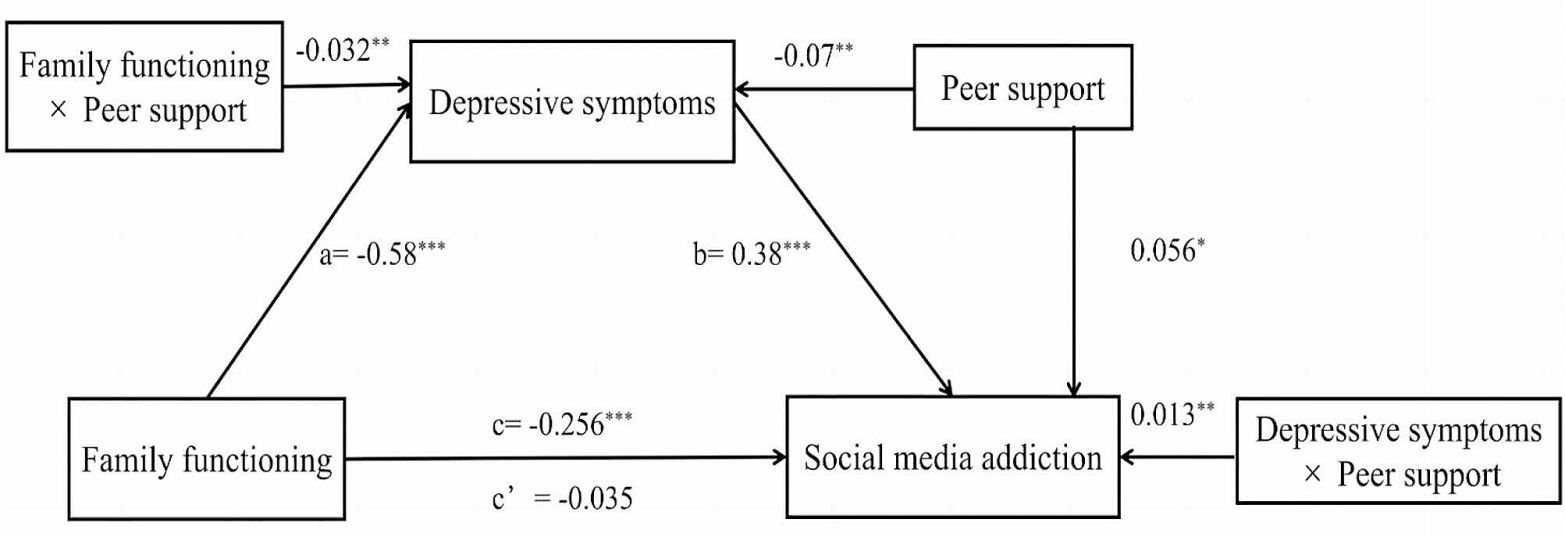
Moderated Mediation effect model. *Note* N = 1840. The variables of age, gender, grade, and major were controlled a, b, c, and c’ are regression coefficients. c = total effect of family functioning on SMA; c’ = direct effect of family functioning on SMA. ^*^p < 0.05, ^**^p < 0.01, ^***^p < 0.001

**Fig. 3:**
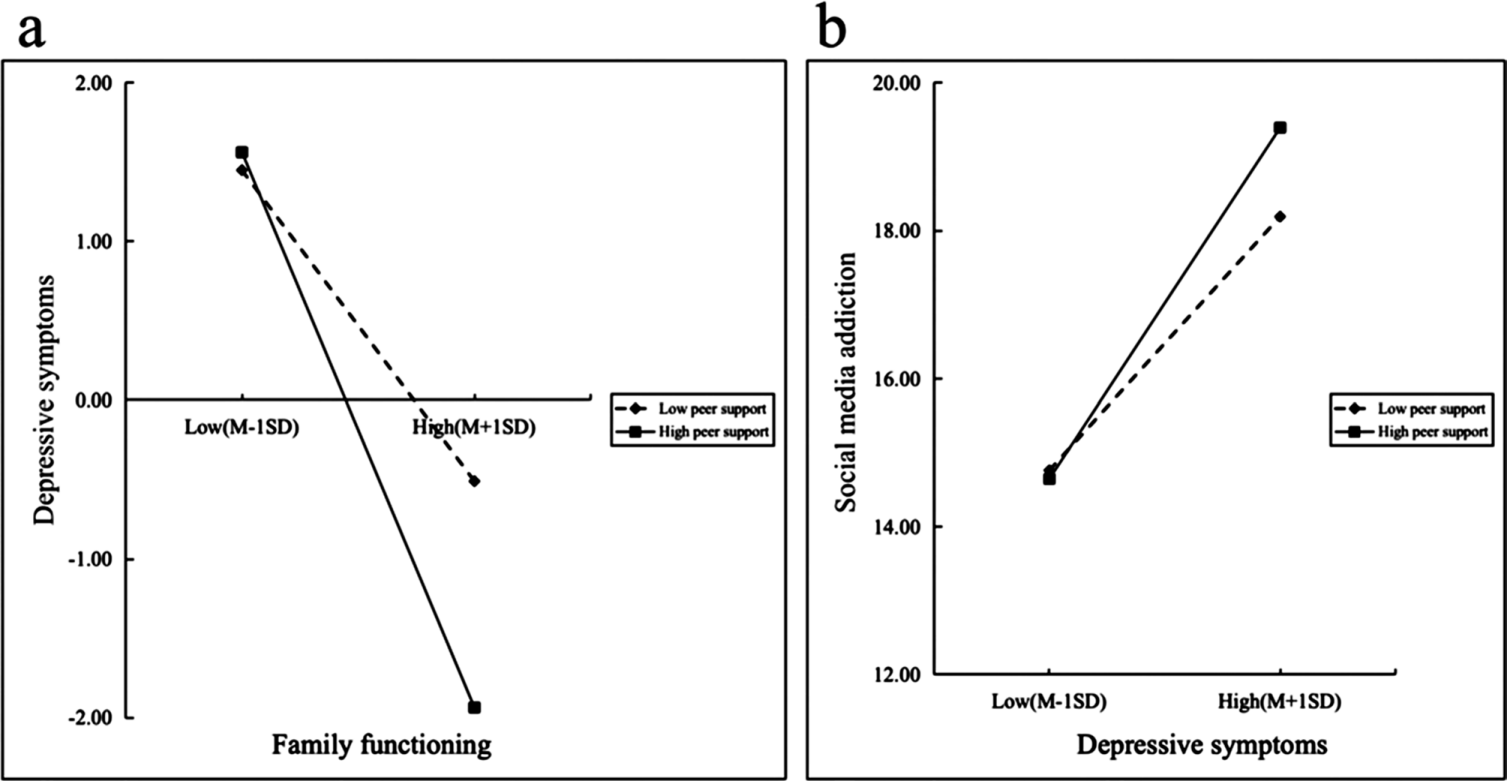
(**a**) The moderating effect of peer support on the relationship between family functioning and depressive symptoms.(**b**) The moderating effect of peer support on the relationship between depressive symptoms and social media addiction. *Note* N=1840. The variables of age, gender, grade, and major were controlled.

Model 59 of the PROCESS was utilized to examine the moderating effects of peer support (see Table 5; Fig. 2) between family functioning (X) and SMA (Y). Model 4a examined the direct moderating effect of peer support on the relationship between family functioning (X) and SMA (Y). Model 4b examined the indirect moderating effect of peer support on the relationship between family functioning (X) and the SMA (Y) via depressive symptoms (M). Basic slope tests were performed to determine the moderating effects of peer support (see Fig. 3). The PROCESS macro provided 95% confidence intervals (CIs) for the Model 59 effect based on 5000 bootstrapped samples. When the 95% CI of the interaction did not contain zero, a significant moderated mediation effect could be established with certainty.

**Table 5:** Moderated mediation regressions of family functioning, peer support, depressive symptoms, and social media addiction

Variables	Model 4a (Depressive symptoms)					Model 4b ( Social media addiction)				
	β	SE	t	LLCI	ULCI	β	SE	t	LLCI	ULCI
Family functioning	-0.55	0.05	-10.34	-0.66	-0.45	-0.07	0.05	-1.28	-0.17	0.04
Depressive symptoms	-	-	-	-	-	0.38	0.02	17.33	0.34	0.43
Peer support	-0.07	0.03	-2.51	-0.12	-0.02	0.06	0.03	2.23	0.01	0.11
Family functioning × peer support	-0.03	0.01	-3.14	-0.05	-0.01	0.01	0.01	0.74	-0.01	0.03
Depressive symptoms × peer support	-	-	-	-	-	0.01	0.004	3.03	0.004	0.02
Age	-0.23	0.16	-1.46	-0.53	-0.08	0.21	0.15	1.42	-0.08	0.49
Gender	-0.87	0.27	-3.24	-1.40	-0.34	0.61	0.25	2.42	0.12	1.10
Grade	0.25	0.18	1.45	-0.09	0.60	-1.00	0.16	-0.59	-0.42	0.22
Major	0.02	0.10	0.22	-0.18	0.22	0.09	0.10	0.98	-0.09	0.28
Constant	5.70	3.01	1.89	-0.20	11.61	11.35	2.81	4.04	5.83	16.86
Adj-R²	0.089					0.166				
F	25.122					39.815				

Note N = 1840. The variables of age, gender, grade, and major were controlled. Abbreviation: LLCI: a lower level

### Common method bias

Common method bias (CMB) could exist when the data are obtained via self-reports. Therefore, the procedure was arranged carefully, and the respondents completed the questionnaire anonymously. Harman’s single-factor test was applied to avoid CMB. The analysis revealed that there were 22 factors with Eigenvalues above 1. The first component explained 14.22% of the variance, which was below the required threshold of 40% [58], indicating that there was no substantial evidence of CMB.

## Results

### Participant characteristics

The descriptive statistics for the variables are displayed in Table 1. Among the 1840 participants, the average age was 20.88 years. There were 535 males (29.08%) and 706 medical students (38.37%) (see Table 1). There were 535 freshmen (29.08%), 359 sophomores (19.51%), and 659 juniors (35.82%). A total of 994 participants were only children. A total of 30.11% had SMA (BSMAS score ≥ 19), 38.80% had a dysfunctional family (APGAR score < 7), and 15.98% had depressive symptoms (PHQ-9 score ≥ 10).

### Correlation analysis of family functioning, depressive symptoms, peer support, and SMA

Partial correlation analysis was conducted for family functioning, depressive symptoms, peer support, and SMA (see Table 2), and demographic factors, including age, gender, grade, and major, were included as control variables. Good family functioning was negatively correlated with depressive symptoms (*r* =-0.27, *p* < 0.001) and SMA (*r* = -0.12, *p* < 0.001). Depressive symptoms were positively correlated with SMA (*r* = 0.40, *p* < 0.001). Peer support had a positive correlation with family functioning (*r* = 0.36, *p* < 0.001) and a negative correlation with depressive symptoms (*r* = -0.16, *p* < 0.001).

### Depressive symptoms as a mediator

Hierarchical multiple regressions were utilized to examine whether depressive symptoms acted as a mediator in the relationship between family functioning and SMA, and the results are shown in Table 3. After adding the control variables, family functioning was negatively related to SMA (*β*= -0.26, 95% CI [-0.35, -0.16]) (see Model 1), supporting Hypothesis 1. After the mediating variable was included, better family functioning negatively predicted depressive symptoms (*β=*-0.58, 95%CI [-0.67, -0.48]) (see Model 2). Depressive symptoms positively predicted SMA (*β* = 0.38, 95%CI[0.34, 0.43]). However, the direct effect of family functioning on SMA was not significant (see Model 3), suggesting that depressive symptoms may fully mediate this relationship. Moreover, Model 4 of the PROCESS macro confirmed that the mediating effect was significant (indirect effect = -0.22, 95%CI [-0.28, -0.17]), suggesting that depressive symptoms mediated this connection (see Table 4), and Hypothesis 2 was confirmed.

### Peer support as a moderator

The results of the moderated mediation regression analysis are displayed in Table 5; Fig. 2. Model 4a showed that the interaction between family functioning and peer support was negatively related to depressive symptoms (*β*= -0.03, 95% CI [-0.05,-0.01]), indicating that peer support may attenuate the relationship between family functioning and depressive symptoms, supporting Hypothesis 3a. Model 4b showed that the interaction effect of depressive symptoms and peer support was positively related to SMA(*β* = 0.01, 95%CI[0.004, 0.02]), indicating that peer support exacerbated the positive effect of depressive symptoms on SMA, supporting Hypothesis 3b. Furthermore, the interaction of family functioning and peer support failed to predict SMA (*β* = 0.01, 95%CI[-0.01, 0.03]), suggesting that peer support did not play a moderating role in the direct relationship between family functioning and SMA, and Hypothesis 3c was rejected.

Finally, simple slope tests were carried out to indirectly determine the moderating effects of peer support on the relationship between family functioning and SMA. Figure 3a showed the conditional direct effects of family functioning (X) on depressive symptoms (M) at different moderator levels, where there was a stronger effect for individuals with higher levels of peer support than for those with lower levels of peer support. It can be inferred from the plots that, with a higher level of peer support and better family functioning, there was an attenuating effect on depressive symptoms. Furthermore, as illustrated in Fig. 3b, there was a stronger effect in the higher level of peer support than the lower-level peer support, and it can be inferred that with a higher level of peer support and more severe depressive symptoms, there was an enhanced effect on SMA.

### Additional analysis

To verify our findings for the moderating effect of peer support, we conducted two regression tests by splitting the sample. First, to test the moderating effect of peer support on the relationship between family functioning and depressive symptoms, we divided the sample into two subsamples using the cutoff score of PHQ-9 [49] to categorize the participants into two groups, including depressive symptoms and non-depressive symptoms groups. The result showed that the relationship between peer support and depressive symptoms was not significant for university students with good family functioning (*β*= -0.032, *p* > 0.05), but was significantly negative for those with dysfunctional family functioning (*β*= -0.138, *p* < 0.001), indicating that peer support could decrease depressive symptoms among university students from dysfunctional families and verifying Hypothesis 3a.

Similarly, we divided the sample into two subsamples using the BSMAS cutoff score [52]to categorize the two groups by testing the moderating role of peer support on the relationship between depressive symptoms and SMA. The results showed that the relationship between peer support and SMA was significantly positive (*β* = 0.274, *p* < 0.001) for university students with depressive symptoms but was not significant for university students without depressive symptoms (*β*= -0.038, *p* > 0.05). To sum up, the results are consistent with those of our main analysis, indicating that peer support could increase SMA among university students with depressive symptoms and verifying Hypothesis 3b. The results from additional regression tests are presented in Supplementary Tables 1 and Table 2.

## Discussion

The main contribution of our study was to explore how depressive symptoms and peer support influence the relationship between family functioning and social media addiction among Chinese university students. According to the conceptual model, our study employed a moderated mediation analysis to reveal three findings. First, good family functioning was significantly related to lower social media addiction. Second, depressive symptoms may function as a mediator in the relationship between family functioning and SMA. Third, the indirect effect of the mediation model was moderated by peer support, as peer support decreased depressive symptoms when family functioning was lower and aggravated social media addiction when university students suffered from depressive symptoms. The results help to elucidate the risk factors and underlying mechanisms of SMA among Chinese university students and provide valuable insight into how family functioning and peer support protect against depressive symptoms and SMA.

### Family functioning and SMA

Our study has confirmed that university students with good family functioning are less likely to suffer from SMA, which aligns with existing studies that have demonstrated the relationship between family functioning and individual development [59]. Additionally, the current findings provided empirical support for the McMaster Family Functioning Model Theory [17] and highlighted the effect of better family functioning on the psychological and behavioral performance of Chinese university students. Good family functioning, including adequate affective communication, may give individuals a vent for negative emotions and prevent them from oversharing or becoming overly dependent on social media [60]. Furthermore, high propinquity interaction between family members, for instance, effective in-person communication, is likely to convey affection and foster family closeness [61], which then contributes to less SMA [23]. Consequently, the criticality of good family functioning in the advancement of university students is brought to light.

### The mediating role of depressive symptoms

Our study also highlighted the mediation effect of depressive symptoms on the relationship between family functioning and SMA. This finding provided evidence for understanding the potential mechanisms underlying the relationship between good family functioning and problematic behavior, which is consistent with prior findings [62]. Better family functioning could be beneficial to superior psychological states and behavioral outcomes [63]. In addition, our findings revealed that depressive symptoms have a positive impact on SMA, in line with studies showing that university students with depressive symptoms are more vulnerable to having SMA [64], supporting the model of compensatory internet use [31]. As such, those who find it difficult to receive comfort from family members are susceptible to suffering from depressive symptoms and may seek insufficient care via social media as a kind of compensation [65]. This study is one of the first to employ such a theory within the family realm by revealing that university students with good family functioning may be conducive to optimizing psychological conditions and improving undesirable behavior.

### The moderating effects of peer support

The most important findings were that peer support had moderated effects on the relationships between family functioning and SMA through depressive symptoms.

On the one hand, peer support could alleviate the relationship between family functioning and depressive symptoms, which was consistent with previous findings that peer support was protective for mental health, especially for individuals lacking support from family members [66]. Our findings highlighted the primary effect model of social support, which posited that peer support may foster the cultivation of favorable psychological attributes and beneficial behavioral patterns [67]. One potential explanation might be that the unfulfilled emotional requirements from the family environment could be slightly compensated for by the relatively higher levels of peer support. Greater family functioning along with a greater level of peer support might enhance the acceptance and understanding of individuals, thus buffering pressure, and improving psychological conditions [68]. Therefore, this study sheds light on the protective role of peer support in relieving negative emotions when family-related factors are at extremely low levels for Chinese university students.

Furthermore, peer support may increase the likelihood of SMA for university students with depressive symptoms, in line with recent findings [45]. According to the compensatory satisfaction theory, appropriate and effective communication is necessary to achieve adequate and timely psychological satisfaction [69]. Owing to the benefits of broadening social networks and furnishing individuals with a platform that enables convenient sharing and communication via social media [70], university students may interact with others online. Consequently, university students who are experiencing depressive symptoms could seek interpersonal interactions by accessing social media, thereby alleviating negative emotions, and attaining satisfaction [71], making them prone to further overindulge in social media and then being addicted to social media.

### Limitations

The limitations of the current study should also be acknowledged. We employed a cross-sectional methodology to discover the relationships between family functioning, peer support, depressive symptoms, and SMA. Although a moderated mediation analysis can predict this association, we could not determine causality. A longitudinal design should be adopted to explore the effect of family functioning on SMA over time in the future. Second, the scope of our study was constrained due to its exclusive execution at Nantong University. In the future, studies should enlarge the sample size and increase the scope of coverage. Third, the survey was based on self-reports. To enhance the accuracy of the results, future research should incorporate unbiased metrics, such as interviews, to obtain objective measures.

This research puts forward several propositions for future studies. First, to verify and examine our model, future studies can explore specific social media addiction (e.g., Facebook [72], YouTube [73]) in our proposed model; In addition, the relationships between family functioning, peer support, depressive symptoms and other types of Internet addiction may also be explored, including gaming and mobile phone addiction; Finally, different types of internet addiction can be assessed using a psychometric sound instrument (e.g., Assessment of Criteria for Specific Internet Use Disorders (ACSID 11) [74].

## Implications

The current study presents a comprehensive framework to clarify the connections between family factors, peer support, psychological factors, and behavioral outcomes. This provides implications for how family functioning and SMA influence depressive symptoms and SMA both theoretically and empirically. Therefore, parents should be more concerned about psychological well-being and problematic behavior by maintaining open lines of communication and exchanging with steady emotional attachment. University staffs should be aware of students’ mental health status and intervene in peer relationships, such as by launching a psychological counseling platform, regularly assessing mental health, and organizing interactive activities.

## Conclusion

To sum up, this study made contributions to the existing research by pinpointing the mediating and moderating factors that establish a connection between family functioning to SMA among Chinese university students. Overall, good family functioning could be related to better mental health and decreased SMA. In addition, a greater level of peer support could decrease individuals’ depressive symptoms when their family functioning is lower and peer support may increase their SMA behaviors with depressive symptoms among Chinese university students. Improving the psychological states and behavioral development of Chinese university students could be prioritized to emphasize the importance of enhancing family functioning and cultivating positive peer support properly.

### Electronic supplementary material

Below is the link to the electronic supplementary material.

Supplementary Material 1

## Data Availability

The data in this paper are from a questionnaire survey. The data may be accessed after obtaining the author’s consent (email:yxgao@ntu.edu.cn).

## Abbreviations

SMA: Social Media Addiction

## References

[1] Feng J, Chen J, Jia L, Liu G (2023). Peer victimization and adolescent problematic social media use: the mediating role of psychological insecurity and the moderating role of family support. Addict Behav.

[2] Xin M, Chen P, Liang Q, Yu C, Zhen S, Zhang W (2021). Cybervictimization and adolescent internet addiction: a Moderated Mediation Model. Int J Environ Res Public Health.

[3] Chen SBRWJL. Enjoy life, work and study encyclopedia, beware of time killers and privacy traps, young and old users of social media surf and control the water China Youth Daily; 2022. http://news.cyol.com/gb/articles/2022-11/25/content_7JedaIeKz.

[4] Tan C-L (2023). Toward an integrated framework for examining the addictive use of smartphones among young adults. Asian J Social Health Behav.

[5] Lin C-Y, Ratan ZA, Pakpour AH (2023). Collection of smartphone and internet addiction. BMC Psychiatry.

[6] Mamede A, Erdem Ö, Noordzij G, Merkelbach I, Kocken P, Denktaş S (2022). Exploring the intersectionality of family SES and gender with psychosocial, behavioural and environmental correlates of physical activity in Dutch adolescents: a cross-sectional study. BMC Public Health.

[7] Andreassen C, Pallesen S (2014). Social Network Site Addiction - An Overview. Curr Pharm Des.

[8] Meng S-Q, Cheng J-L, Li Y-Y, Yang X-Q, Zheng J-W, Chang X-W (2022). Global prevalence of digital addiction in general population: a systematic review and meta-analysis. Clin Psychol Rev.

[9] Chi X, Lin L, Zhang P (2016). Internet addiction among College students in China: Prevalence and Psychosocial correlates. Cyberpsychol Behav Soc Netw.

[10] Al-Yafi K, El-Masri M, Tsai R (2018). The effects of using social network sites on academic performance: the case of Qatar. J Enterp Inform Manage.

[11] Zou L, Wu X, Tao S, Xu H, Xie Y, Yang Y et al. Mediating effect of Sleep Quality on the Relationship between Problematic Mobile phone use and depressive symptoms in College Students. Front Psychiatry. 2019;10. 10.3389/fpsyt.2019.00822 PMC6865206 31798473

[12] Alimoradi Z, Broström A, Potenza MN, Lin C-Y, Pakpour AH (2024). Associations between behavioral addictions and Mental Health concerns during the COVID-19 pandemic: a systematic review and Meta-analysis. Curr Addict Rep.

[13] Marin MG, Nuñez X, de Almeida RMM (2021). Internet addiction and attention in adolescents: a systematic review. Cyberpsychol Behav Soc Netw.

[14] Bronfenbrenner U (2000). Ecological systems theory. Encyclopedia of psychology.

[15] Tereshchenko SY (2023). Neurobiological risk factors for problematic social media use as a specific form of internet addiction: a narrative review. World J Psychiatry.

[16] Casale S, Banchi V (2020). Narcissism and problematic social media use: a systematic literature review. Addict Behav Rep.

[17] Miller IW, Ryan CE, Keitner GI, Bishop DS, Epstein NB (2000). The McMaster Approach to families: theory, assessment, treatment and research. J Fam Ther.

[18] Sabah A, Aljaberi MA, Hajji J, Fang C-Y, Lai Y-C, Lin C-Y (2023). Family Communication as a mediator between Family Resilience and Family Functioning under the Quarantine and COVID-19 pandemic in Arabic Countries. Children.

[19] Zhang X, Zhao M, Li J, Shi L, Xu X, Dai Q (2019). Associations between family cohesion, adaptability, and functioning of patients with bipolar disorder with clinical syndromes in Hebei, China. J Int Med Res.

[20] Nielsen P, Favez N, Rigter H (2020). Parental and family factors Associated with problematic gaming and problematic internet use in adolescents: a systematic literature review. Curr Addict Rep.

[21] Yu L, Shek DTL (2013). Internet addiction in Hong Kong adolescents: a three-year longitudinal study. J Pediatr Adolesc Gynecol.

[22] Terres-Trindade M, Mosmann CP (2015). Discriminant Profile of Young Internet dependents: the role of Family relationships. Paidéia (Ribeirão Preto).

[23] Simpson EG, Backman A, Ohannessian CM (2023). Family Functioning and Social Media Use in Early Adolescence. J Child Fam Stud.

[24] Azagba S, Ebling T, Korkmaz A (2024). Social media and e-cigarette use: the mediating role of mental health conditions. J Affect Disord.

[25] Elhai JD, Yang H, Fang J, Bai X, Hall BJ (2020). Depression and anxiety symptoms are related to problematic smartphone use severity in Chinese young adults: fear of missing out as a mediator. Addict Behav.

[26] Yoon S, Kleinman M, Mertz J, Brannick M (2019). Is social network site usage related to depression? A meta-analysis of Facebook–depression relations. J Affect Disord.

[27] Chentsova VO, Bravo AJ, Mezquita L, Pilatti A, Hogarth L (2023). Internalizing symptoms, rumination, and problematic social networking site use: a cross national examination among young adults in seven countries. Addict Behav.

[28] Nam B, Kim JY, DeVylder JE, Song A (2016). Family functioning, resilience, and depression among North Korean refugees. Psychiatry Res.

[29] Wang M, He N, Xu Q, Yue Y, Li M, Lin J (2022). Childhood emotional neglect and problematic social media use among Chinese adolescents: a moderated mediation model. Addict Behav.

[30] Lecompte V, Moss E, Cyr C, Pascuzzo K (2014). Preschool attachment, self-esteem and the development of preadolescent anxiety and depressive symptoms. Attach Hum Dev.

[31] Kardefelt-Winther D (2014). A conceptual and methodological critique of internet addiction research: towards a model of compensatory internet use. Comput Hum Behav.

[32] Kabasakal Z (2015). Life satisfaction and family functions as-predictors of problematic internet use in university students. Comput Hum Behav.

[33] Alfaya MA, Abdullah NS, Alshahrani NZ, Alqahtani AAA, Algethami MR, Al Qahtani ASY (2023). Prevalence and Determinants of Social Media Addiction among medical students in a selected University in Saudi Arabia: a cross-sectional study. Healthcare.

[34] Landers GM, Zhou M (2011). An analysis of relationships among peer support, Psychiatric hospitalization, and Crisis stabilization. Community Ment Health J.

[35] Pavarini G, Reardon T, Hollowell A, Bennett V, Lawrance E, Brooks-Hall E (2023). Online peer support training to promote adolescents’ emotional support skills, mental health and agency during COVID-19: Randomised controlled trial and qualitative evaluation. Eur Child Adolesc Psychiatry.

[36] Gunuc S, Dogan A (2013). The relationships between Turkish adolescents’ internet addiction, their perceived social support and family activities. Comput Hum Behav.

[37] Choo H, Chng GS, Gentile DA, Lau SPC (2021). The role of peer support in the growth trajectory of pathological internet use among Youth: a protective factor. Cyberpsychol Behav Soc Netw.

[38] Kuss DJ, Griffiths MD, Binder JF (2013). Internet addiction in students: prevalence and risk factors. Comput Hum Behav.

[39] Hu X, Song Y, Zhu R, He S, Zhou B, Li X (2022). Understanding the impact of emotional support on mental health resilience of the community in the social media in Covid-19 pandemic. J Affect Disord.

[40] Cohen S, Wills TA (1985). Stress, social support, and the buffering hypothesis. Psychol Bull.

[41] Cooper RE, Saunders KRK, Greenburgh A, Shah P, Appleton R, Machin K (2024). The effectiveness, implementation, and experiences of peer support approaches for mental health: a systematic umbrella review. BMC Med.

[42] Merritt DH, Snyder SM (2015). Correlates of optimal behavior among child welfare-involved children: perceived school peer connectedness, activity participation, social skills, and peer affiliation. Am J Orthopsychiatry.

[43] Wang E, Zhang J, Peng S, Zeng B. The association between family function and adolescents’ depressive symptoms in China: a longitudinal cross-lagged analysis. Front Psychiatry. 2021;12. 10.3389/fpsyt.2021.744976 PMC8718401 34975563

[44] Chen Q, Song Y, Huang Y, Li C (2023). The interactive effects of family violence and peer support on adolescent depressive symptoms: the mediating role of cognitive vulnerabilities. J Affect Disord.

[45] Huang L, Zhang J, Duan W, He L (2023). Peer relationship increasing the risk of social media addiction among Chinese adolescents who have negative emotions. Curr Psychol.

[46] The Family APGAR (1978). A proposal for a family function test and its use by Physicians. J Fam Pract.

[47] Hu Y, Yang Z, Li Y, Xu Y, Zhou X, Guo N. Anxiety symptoms and Associated factors among chronic low back Pain patients in China: a cross-sectional study. Front Public Health. 2022;10. 10.3389/fpubh.2022.878865 PMC9114483 35602156

[48] Kroenke K, Spitzer RL (2002). The PHQ-9: a New Depression Diagnostic and Severity measure. Psychiatr Ann.

[49] Lin W, Wu B, Chen B, Zhong C, Huang W, Yuan S (2021). Associations of COVID-19 related experiences with maternal anxiety and depression: implications for mental health management of pregnant women in the post-pandemic era. Psychiatry Res.

[50] Dong W, Xiao-hui X, Xian-bo W (2012). The healthy lifestyle scale for University students: development and psychometric testing. Aust J Prim Health.

[51] Andreassen CS, Pallesen S, Griffiths MD (2017). The relationship between addictive use of social media, narcissism, and self-esteem: findings from a large national survey. Addict Behav.

[52] Leung H, Pakpour AH, Strong C, Lin Y-C, Tsai M-C, Griffiths MD (2020). Measurement invariance across young adults from Hong Kong and Taiwan among three internet-related addiction scales: Bergen Social Media Addiction Scale (BSMAS), Smartphone Application-based addiction scale (SABAS), and internet gaming disorder scale-short form (IGDS-SF9) (study part A). Addict Behav.

[53] Yam C-W, Pakpour AH, Griffiths MD, Yau W-Y, Lo C-LM, Ng JMT (2019). Psychometric testing of three Chinese online-related addictive Behavior instruments among Hong Kong University students. Psychiatr Q.

[54] Schivinski B, Brzozowska-Woś M, Stansbury E, Satel J, Montag C, Pontes HM. Exploring the role of Social Media Use motives, Psychological Well-Being, Self-Esteem, and affect in problematic Social Media Use. Front Psychol. 2020;11. 10.3389/fpsyg.2020.617140 PMC7772182 33391137

[55] Boer M, van den Eijnden RJJM, Boniel-Nissim M, Wong S-L, Inchley JC, Badura P (2020). Adolescents’ intense and problematic Social Media Use and their Well-being in 29 countries. J Adolesc Health.

[56] Chen Y, Liu X, Chiu DT, Li Y, Mi B, Zhang Y (2022). Problematic social media use and depressive outcomes among College students in China: observational and experimental findings. Int J Environ Res Public Health.

[57] Baker I, Marzouqa N, Yaghi BN, Adawi SO, Yousef S, Sabooh TN (2021). The impact of information sources on COVID-19-Related knowledge, attitudes, and practices (KAP) among University students: a nationwide cross-sectional study. Int J Environ Res Public Health.

[58] Li G-R, Sun J, Ye J-N, Hou X-H, Xiang M-Q. Family functioning and mobile phone addiction in university students: mediating effect of loneliness and moderating effect of capacity to be alone. Front Psychol. 2023;14. 10.3389/fpsyg.2023.1076852 PMC9947282 36844342

[59] Assunção RS, Costa P, Tagliabue S, Mena Matos P (2017). Problematic Facebook Use in adolescents: associations with parental attachment and alienation to peers. J Child Fam Stud.

[60] Qian L, Wang D, Jiang M, Wu W, Ni C. The impact of Family Functioning on College Students’ loneliness: Chain-Mediating effects of Core self-evaluation and problematic mobile phone use. Front Psychol. 2022;13. 10.3389/fpsyg.2022.915697 PMC9296845 35874368

[61] Korzenny F (1978). A theory of electronic propinquity. Communic Res.

[62] Zheng B, Yu Y, Zhu X, Hu Z, Zhou W, Yin S (2020). Association between family functions and antenatal depression symptoms: a cross-sectional study among pregnant women in urban communities of Hengyang city, China. BMJ Open.

[63] Pan Y, Yang Z, Han X, Qi S (2021). Family functioning and mental health among secondary vocational students during the COVID-19 epidemic: a moderated mediation model. Pers Individ Dif.

[64] Ryan RM, Deci EL (2000). The darker and brighter sides of human existence: Basic Psychological needs as a Unifying Concept. Psychol Inq.

[65] Liu Q-Q, Yang X-J, Hu Y-T, Zhang C-Y, Nie Y-G (2020). How and when is family dysfunction associated with adolescent mobile phone addiction? Testing a moderated mediation model. Child Youth Serv Rev.

[66] McLeod J, Davis CG (2023). Community peer support among individuals living with spinal cord injury. J Health Psychol.

[67] Henry PJ. Social Psychology: Handbook of Basic Principles (2nd edition) – Edited by A. Kruglanski and E. T. Higgins. Polit Psychol. 2008;29:792–7.

[68] Ouyang M, Gui D, Cai X, Yin Y, Mao X, Huang S et al. Stressful life events and subjective well-being in Vocational School Female adolescents: the mediating role of Depression and the moderating role of Perceived Social Support. Front Psychol. 2021;11. 10.3389/fpsyg.2020.603511 PMC7933236 33679496

[69] Liu Q-X, Fang X-Y, Wan J-J, Zhou Z-K (2016). Need satisfaction and adolescent pathological internet use: comparison of satisfaction perceived online and offline. Comput Hum Behav.

[70] Dwyer C. Digital Relationships in the MySpace Generation: Results From a Qualitative Study. In: 2007 40th Annual Hawaii International Conference on System Sciences (HICSS’07). IEEE; 2007. pp. 19–19.

[71] Wang T, Mu W, Li X, Gu X, Duan W (2022). Cyber-ostracism and wellbeing: a moderated mediation model of need satisfaction and psychological stress. Curr Psychol.

[72] Mamun MA, Al, Griffiths MD (2019). The association between Facebook addiction and depression: a pilot survey study among Bangladeshi students. Psychiatry Res.

[73] Pakpour AH, Jafari E, Zanjanchi F, Potenza MN, Lin C-Y (2023). The YouTube Addiction Scale: psychometric evidence for a new instrument developed based on the Component Model of Addiction. Int J Ment Health Addict.

[74] Huang Y-T, Ruckwongpatr K, Chen J-K, Pakpour AH, Siaw Y-L, Nadhiroh SR (2024). Specific Internet Disorders in University Students in Taiwan and Hong Kong: Psychometric Properties with Invariance Testing for the traditional Chinese version of the Assessment of Criteria for Specific Internet-Use disorders (ACSID-11). Int J Ment Health Addict.

