# Supplementary material for: The relationship between family functioning and social media addiction among university students: a moderated mediation model of depressive symptoms and peer support
Source: BMC Psychol. 2024 Jun 10;12:341. doi: 10.1186/s40359-024-01818-2 (PMC11165749; doi:10.1186/s40359-024-01818-2)
Supplement: Supplementary file 1 — Supplementary Material 1 [file 40359_2024_1818_MOESM1_ESM.docx]

**Supplementary 1**

The moderating effect of peer support on the relationship between family functioning and depressive symptoms.

| Variables | Family dysfunction | | | Good family functioning | | |
| --- | --- | --- | --- | --- | --- | --- |
|  | *β* | SE | *p* | *β* | SE | *p* |
| Peer support | -0.138^***^ | 0.031 | <0.001 | -0.032 | 0.050 | 0.517 |
| Age | -0.067 | 0.205 | 0.743 | -0.374 | 0.251 | 0.136 |
| Gender | -0.916^**^ | 0.342 | <0.01 | -0.705 | 0.448 | 0.116 |
| Grade | 0.093 | 0.226 | 0.680 | 0.403 | 0.288 | 0.161 |
| Major | 0.073 | 0.124 | 0.559 | -0.256 | 0.180 | 0.887 |
| Constant | 10.424^**^ | 4.008 | <0.01 | 15.704^**^ | 4.892 | <0.01 |

Note: N=1840. The variables of age, gender, grade, and major were controlled.

^*^*p*<0.05, ^**^*p*<0.01, ^***^*p*<0.001.

**Supplementary 2**

The moderating effect of peer support on the relationship between depressive symptoms and social media addiction

| Variables | Depressive symptoms | | | Non-Depressive symptoms | | |
| --- | --- | --- | --- | --- | --- | --- |
|  | *β* | SE | *p* | *β* | SE | *p* |
| Peer support | 0.274^***^ | 0.067 | <0.001 | -0.038 | 0.026 | 0.141 |
| Age | 0.234 | 0.438 | 0.594 | 0.180 | 0.160 | 0.260 |
| Gender | -0.035^**^ | 0.659 | 0.970 | 0.586^*^ | 0.283 | <0.05 |
| Grade | 0.003 | 0.468 | 0.994 | -0.109 | 0.180 | 0.545 |
| Major | 0.125 | 0.279 | 0.656 | 0.057 | 0.104 | 0.582 |
| Constant | 9.146 | 8.507 | 0.283 | 12.235^***^ | 3.136 | <0.001 |

Note: N=1840. The variables of age, gender, grade, and major were controlled.

^*^*p*<0.05, ^**^*p*<0.01, ^***^*p*<0.001.
